# Socioeconomic inequalities in the continuum of care for maternal health in an upper-middle-income country: the case of Mexico

**DOI:** 10.1590/0102-311XEN232925

**Published:** 2026-08-21

**Authors:** Leticia Suárez-López, Julio César Montañez-Hernández, Nadia Cerecer-Ortiz, Celia Hubert, Ileana Heredia-Pi, Aremis Villalobos, Leticia Avila-Burgos

**Affiliations:** 1 Centro de Investigación en Salud Poblacional, Instituto Nacional de Salud Pública, Cuernavaca, México.; 2 Centro de Investigación en Sistemas de Salud, Instituto Nacional de Salud Pública, Cuernavaca, México.

**Keywords:** Continuity of Patiente Care, Maternal Health, Socioeconomic Disparities, Continuidad de la Atención al Paciente, Salud Materna, Disparidades Socioeconómicas, Continuidade da Assistência ao Paciente, Saúde Materna, Disparidades Socioeconômicas

## Abstract

This cross-sectional study aimed to identify the magnitude of socioeconomic inequalities in the continuum of care for maternal health according to socioeconomic status and the contribution of sociodemographic factors to this inequality. We used data from the *Mexican National Continuous Health and Nutrition Survey* (2021-2023), which included 2,846 women who had recently given birth. We assessed both independent and conditional coverage indicators across the maternal health care continuum. We analyzed the association with socioeconomic status using logistic regression and measured inequality with the concentration index and Wagstaff-type decomposition analysis. The prevalence of the continuum of care for maternal health was 37.3% (95%CI: 34.6-40.1). Women affiliated with Social Security health services (OR = 1.37, 95%CI: 1.1-1.8), those whose children were born before the pandemic (OR = 1.84, 95%CI: 1.4-2.4), and non-Indigenous women (OR = 2.08, 95%CI: 1.2-3.6) were more likely to receive the continuum of care. The estimated inequality was concentration index = 0.098 (p < 0.05), indicating a pro-rich distribution. The main contributing factors were non-Indigenous status (30%), affiliation with Social Security (31.1%), and pre-pandemic period (9.6%). Our analysis identified the factors contributing to inequalities across various social groups. By decomposition analysis, we found that access to Social Security services, particularly for vulnerable populations such as Indigenous women, plays a crucial role in reducing these disparities.

## Abstract (es)

Este estudio transversal tuvo el objetivo de identificar la magnitud de las desigualdades socioeconómicas en el continuo de la atención a la salud materna según el estatus socioeconómico y la contribución de los factores sociodemográficos a dicha desigualdad. Utilizamos los datos de la *Encuesta Nacional Continua de Salud y Nutrición* (2021-2023) de México, que incluyó a 2.846 mujeres que habían dado a luz recientemente. Evaluamos los indicadores de cobertura tanto independientes como condicionales a lo largo del continuo. Analizamos la asociación con el estatus socioeconómico mediante regresión logística, y medimos la desigualdad utilizando el índice de concentración y el análisis de descomposición de tipo Wagstaff. La prevalencia del continuo de atención a la salud materna fue del 37,3% (IC95%: 34,6-40,1). Las mujeres afiliadas a los servicios de salud de la Seguridad Social (OR = 1,37, IC95%: 1,1-1,8), las personas cuyos hijos nacieron durante el período prepandémico (OR = 1,84, IC95%: 1,4-2,4), y las mujeres no indígenas (OR = 2,08, IC95%: 1,2-3,6) presentaron una mayor probabilidad de acceder al continuo de atención. La desigualdad estimada fue índice de concentración = 0,098 (p < 0,05), lo que indica una distribución favorable a los ricos. Los principales factores contribuyentes fueron la condición de no indígena (el 30%), la afiliación a la Seguridad Social (el 31,1%) y el periodo previo a la pandemia (9,6%). Nuestro análisis identificó los factores que contribuyen a las desigualdades entre diversos grupos sociales. A través del análisis de descomposición, observamos que el acceso a los servicios de la Seguridad Social, principalmente para poblaciones vulnerables como las mujeres indígenas, desempeña un papel fundamental en la reducción de estas disparidades.

## Abstract (pt)

Este estudo transversal buscou identificar a magnitude das desigualdades socioeconômicas no *continuum* de cuidado à saúde materna segundo o *status* socioeconômico e a contribuição dos fatores sociodemográficos para tal desigualdade. Utilizamos dados da *Pesquisa Nacional Contínua de Saúde e Nutrição* (2021-2023) do México, que incluiu 2.846 mulheres que haviam dado à luz recentemente. Avaliamos indicadores de cobertura tanto independentes quanto condicionais ao longo do *continuum*. Analisamos a associação com o status socioeconômico usando regressão logística, e medimos a desigualdade com o índice de concentração e a análise de decomposição do tipo Wagstaff. A prevalência do *continuum* de cuidados à saúde materna foi de 37,3% (IC95%: 34,6-40,1). Mulheres afiliadas aos serviços de saúde da Previdência Social (OR = 1,37, IC95%: 1,1-1,8), aqueles cujos filhos nasceram durante o período pré-pandemia (OR = 1,84, IC95%: 1,4-2,4), e mulheres não indígenas (OR = 2,08, IC95%: 1,2-3,6) apresentaram maior probabilidade de acessar o *continuum* de cuidados. A desigualdade estimada foi índice de concentração = 0,098 (p < 0,05), indicando uma distribuição favorável aos ricos. Os principais fatores contribuintes foram o *status* de não indígena (30%), a filiação à Previdência Social (31,1%) e o período pré-pandemia (9,6%). Nossa análise identificou os fatores que contribuem para as desigualdades entre vários grupos sociais. Por meio da análise de decomposição, descobrimos que o acesso aos serviços de Previdência Social, especialmente para populações vulneráveis como mulheres indígenas, desempenha um papel crucial na redução dessas disparidades.

## Introduction

Motherhood represents a significant stage in women’s lives that should ideally be a positive experience; for some, however, it is marked by illness and even death due to complications during pregnancy, childbirth, or the postpartum period. Most preventable maternal deaths occur in low- and middle-income countries ^1^. This situation has prompted a global shift in care, emphasizing compliance with the continuum of care for maternal health, which encompasses pregnancy, childbirth, and the postpartum period. The continuum of care-maternal health approach includes essential interventions that should be performed during each maternity phase to ensure a positive and healthy experience. Assessments can measure either the implementation of individual interventions at each phase (independent coverage indicators) or the completion of all required interventions across the full maternity cycle (conditional coverage indicators), with the latter offering a closer approximation to comprehensive maternal health care ^2^ ^,^ ^3^. The continuum of care-maternal health stresses the importance of ensuring that all women have access to quality professional care during this stage of life, and that their newborns receive essential services to support healthy motherhood and improve child survival prospects ^4^.

Assessments using independent coverage indicators have reported an improvement in global access to maternal health care. However, when conditional coverage estimates are applied, the level of coverage achieved across the entire continuum of care-maternal health decreases significantly, revealing how independent indicators mask the true extent of comprehensive and integrated care ^5^. Moreover, inequalities persist regarding compliance with the continuum of care-maternal health: women in low-income countries, and more generally those affected by socioeconomic disadvantage, experience the lowest coverage rates ^5^ ^,^ ^6^. In 2023, 70% of pregnant women worldwide attended at least four antenatal visits, compared with 50% in low-income countries and 91% in upper-middle-income countries. Between 2017 and 2023, hospital-based childbirth care reached 79.1% globally, with a 15.5-percentage-point gap between low- and high-income countries (62.4% vs. 94.5%). In 2023, skilled birth attendance reached 86.3% globally, with a 22-percentage-point gap (75.5% vs. 97.9%). Postpartum care within two days of parturition was provided to 68.9% of women worldwide in 2023, with a 33-percentage-point gap between low- and high-income countries (54.5% vs. 87.8%) ^7^.

Research regarding the continuum of care-maternal health shows that factors such as limited income ^8^ ^,^ ^9^ ^,^ ^10^, young age ^11^ low educational level of women ^8^ ^,^ ^9^ ^,^ ^12^ or their partners ^10^, residence in a rural or marginalized area ^8^ ^,^ ^9^ ^,^ ^12^, ethnicity ^13^, religion ^8^ ^,^ ^9^ and restricted decision-making power ^8^, are associated with a reduced likelihood of using quality maternal health services. Quality in this context encompasses not only access and frequency of care, but also its content and, crucially, the continuity of the care process ^2^ ^,^ ^3^.

In Mexico, over the past two decades, various initiatives and increased investment in sexual and reproductive health services - particularly for socioeconomically disadvantaged women ^14^ - have more than doubled continuum of care-maternal health coverage ^4^. Despite these advances, inequalities persist, indicating the existence of discrimination in the continuum of care, primarily by age ^15^ and ethnicity ^13^, which may stem from underlying discriminatory practices both in communities and within health institutions.

In general, the poorest and most marginalized women have benefited least from advances in maternal health care coverage ^1^ ^,^ ^5^ ^,^ ^9^ ^,^ ^10^ ^,^ ^16^. The Guttmacher-Lancet Commission has called for expanded access to a comprehensive package of sexual and reproductive health interventions, prioritizing vulnerable populations ^17^. The equitable provision of timely and qualified maternal health services is recognized as an effective strategy for achieving the Sustainable Development Goals (targets 3.1-3.2) ^1^ ^,^ ^6^ as well as for ensuring safe motherhood and improving newborn survival and health ^1^. Monitoring inequalities in access to sexual and reproductive health services is therefore essential to improve service delivery for disadvantaged groups.

In this context, it is crucial to analyze inequalities in maternal health care across groups defined by socioeconomic status. Although several studies in Mexico have examined disparities in adequate maternal health care ^4^ ^,^ ^11^ ^,^ ^13^ ^,^ ^16^ ^,^ ^18^, few have assessed the extent to which socioeconomic factors account for these inequalities. Accordingly, this study aimed to identify the magnitude of inequalities in the continuum of care-maternal health by socioeconomic status, and to determine the contribution of sociodemographic factors.

### Context of the Mexican health system

Mexico’s public health system is organized into two main subsystems. The contributory Social Security subsystem, comprising several Social Security institutions that have remained largely unchanged since their creation, provides comprehensive care to salaried workers and their families (50.7% of the population). The second subsystem, financed through general taxation, serves individuals without formal employment and without Social Security coverage (48.7% of the population) ^19^. Since 2019, care for the population without Social Security coverage has undergone a process of recentralization. With the creation of the Institute of Health for Well-being (INSABI, acronym in Spanish) and later IMSS-Well-being (IMSS-B, acronym in Spanish), 71% of states in this federalized model shifted their first-level care facilities, public hospitals, and personnel to federal control. As a result, the Federal Government assumed responsibility for providing free health services to those not covered by Social Security. These changes reduced and weakened state-level autonomy, limiting adaptation to local contexts - particularly in Indigenous and rural areas - and increased heterogeneity in access to and quality of services ^20^ ^,^ ^21^.

Although maternal care for women without Social Security coverage is conceived as a comprehensive model that prioritises respectful, person-centred care and intercultural childbirth ^22^, structural challenges persist. These include historical underfunding ^14^ - in 2023, per capita expenditure was USD 567 for the population with Social Security coverage compared to USD 343 for the uninsured ^23^ - as well as insufficient infrastructure, workforce, and availability of medicines. Recent reforms have also been associated with a reduction in effective coverage, disproportionately affecting poor and Indigenous populations ^20^ ^,^ ^24^.

## Material and methods

We conducted a repeated cross-sectional study based on the 2021, 2022, and 2023 waves of the *Mexican National Continuous Health and Nutrition Survey* (ENSANUT, acronym in Spanish). These probabilistic, stratified, two-stage, cluster-design assessments were conducted in households across all 32 Mexican states. Samples were representative at the national and state levels and disaggregated into urban and rural strata. ENSANUT methodology has been described in detail in previous publications ^25^ ^,^ ^26^ ^,^ ^27^. Ethical approval was obtained from the Research Ethics Committees (CI: 1750, 1807, 1865, respectively) of the National Institute of Public Health. Data are public and available at https://ensanut.insp.mx/.

**Figure 1:**
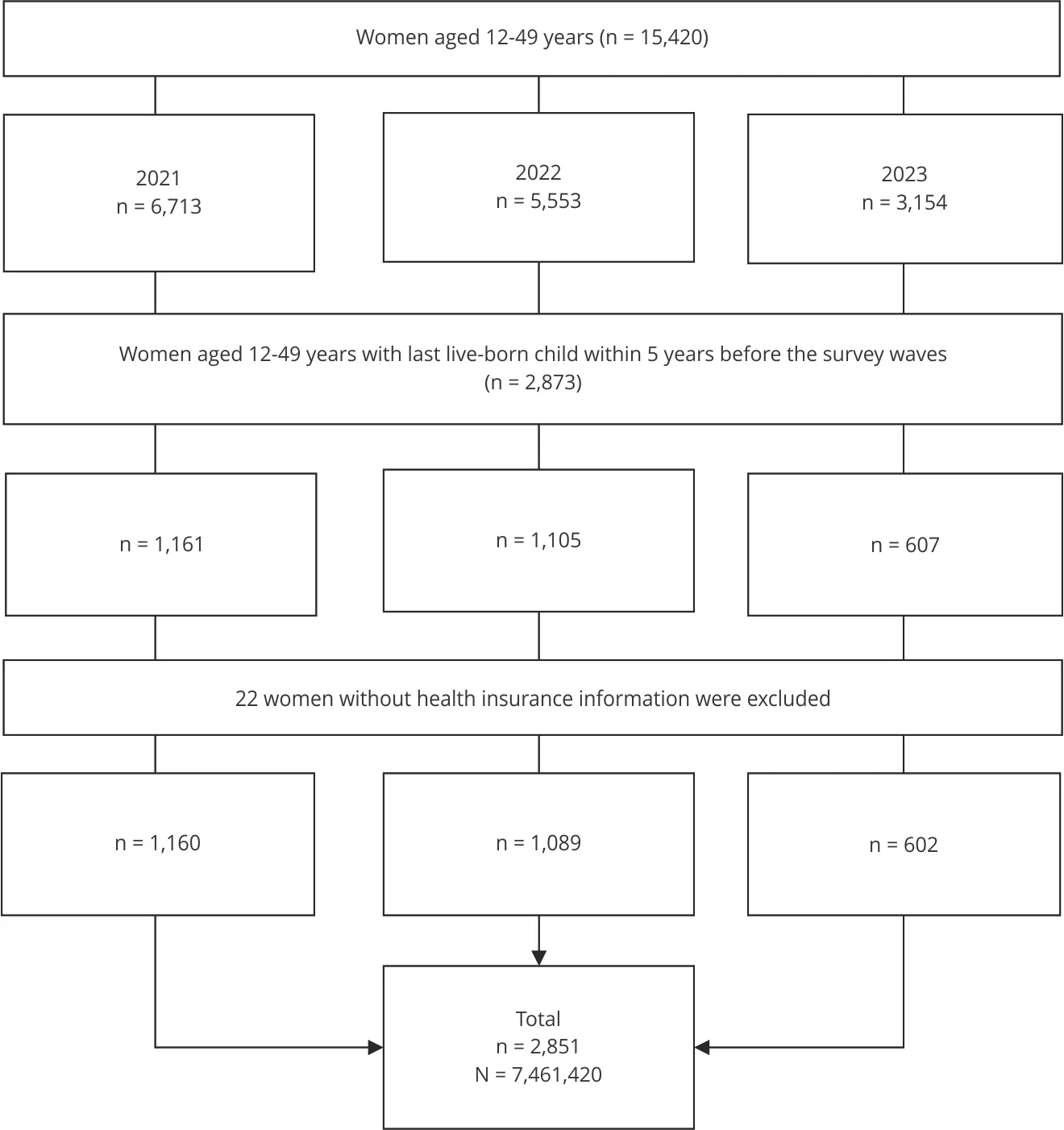
Flowchart of the study.

We analyzed the survey sections on sociodemographic, household, and sexual and reproductive health characteristics. The sample included women aged 12 to 49 years whose last pregnancy occurred within five years prior to each survey wave and resulted in a live birth. After excluding 0.006% of cases with incomplete data for the variables of interest, 2,846 women were retained in the analysis (Figure 1).

### Outcome variable

Based on previous studies ^3^ ^,^ ^4^ ^,^ ^11^ ^,^ ^18^ and the official Mexican guidelines ^28^, we analyzed continuum of care-maternal health using six independent binary (yes = 1/no = 0) indicators corresponding to the various phases of maternity. Antenatal care was assessed by four indicators: (i) receipt of antenatal care from skilled health personnel (physician or nurse); (ii) initiation of care within the first eight weeks of pregnancy (timely antenatal care); (iii) completion of at least five antenatal visits (frequent antenatal care); and (iv) receipt of ≥ 75% of recommended antenatal care procedures (adequate content) ^4^ ^,^ ^11^ ^,^ ^19^ ^,^ ^29^. Childbirth care was measured as hospital-based parturition (institutional childbirth), and postpartum care as at least one consultation within the first week after birth.

Subsequently, we created six binary variables indicating sequential access to interventions (i)-(vi) along the entire antenatal-postnatal continuum. These conditional coverage indicators were based on the coverage cascade principle; that is, receipt of care for each indicator was conditional on receipt of care for the preceding indicator(s) ^3^ ^,^ ^4^ ^,^ ^30^. Accordingly, the proportion of women receiving continuous care was defined as those who received all six interventions across the antenatal to postnatal cycle.

### Socioeconomic status

Women included in the study were stratified by socioeconomic status using the Well-being Conditions Index within the ENSANUT framework. This index, described elsewhere ^31^, is constructed from information on housing characteristics and household assets, including construction materials for floors, walls, and roofs; number of rooms used for sleeping; water availability; car ownership; household items (e.g., refrigerator, washing machine, microwave, stove, boiler); and electrical appliances (e.g., TV, cable, radio, cell phone, computer). Principal components analysis was applied, and the first component was selected, accounting for 45.3% of the total variance. The Index was categorized into tertiles to analyze the populations with lower levels of well-being relative to more advantaged groups (tertile 3).

### Sociodemographic characteristics of women and their households

The sociodemographic characteristics of participants were assessed at the individual and household levels. At the individual level, we considered maternal age at childbirth (0 = adolescents < 20 years, 1 = adults 20-49 years); education (0 = primary or less, 1 = secondary or higher); marital status (0 = without partner, 1 = with partner); primiparity (0 = yes, 1 = no); and health insurance status (0 = no Social Security, 1 = affiliated). Household-level variables included socioeconomic status (0 = tertile 1, 1 = tertile 2, 2 = tertile 3), Indigenous status, and place of residence (0 = rural, 1 = urban). Indigenous status followed the definition of the National Commission for the Development of Indigenous Peoples, which considers individuals as Indigenous if they speak an Indigenous language or belong to a household where the head, spouse, or direct ascendant reports speaking an indigenous language ^32^. Localities with < 2,500 inhabitants were classified as rural, and those with ≥ 2,500 inhabitants as urban. We also included a dichotomous variable for the year of birth of the last live-born child: 1 = born before March 23, 2020 (pre-pandemic, corresponding to the official COVID-19 emergency declaration in Mexico ^33^), and 0 = born thereafter (pandemic period).

### Analytical strategy

Estimates for both independent and conditional coverage indicators along the continuum of care-maternal health were generated, and their association with socioeconomic status was assessed using chi-square tests. A logistic regression model was then fitted to explore the association between continuum of care-maternal health and women’s sociodemographic characteristics, with model goodness of fit assessed by the Hosmer-Lemeshow test.

### Estimating inequality in the continuum of care for maternal health

Since continuum of care-maternal health was defined as a dichotomous indicator, we used the concentration index proposed by Wagstaff ^(^ ^34^ ^)^ for this type of variable to estimate inequality along continuum of care-maternal health according to socioeconomic status. In brief, the concentration index is derived from the concentration curve, which plots the cumulative percentage of the outcome variable (vertical axis) against the cumulative percentage of the population ranked by socioeconomic status (horizontal axis). Under perfect equality, the concentration curve coincides with the 45-degree line of equality. When inequality exists, the concentration index equals twice the area between the concentration curve and the equality line ^35^, with values ranging from -1 to 1. Taking continuum of care-maternal health coverage as the outcome variable, a positive concentration index was undesirable because it would have indicated that coverage favored women with higher socioeconomic status (pro-rich concentration), while a negative concentration interval would have denoted concentration among lower socioeconomic status groups (pro-poor concentration). A concentration index of zero would have implied equality in coverage across socioeconomic status. Higher positive values would have favored women with greater economic resources, therefore signaling wider inequality, while negative values were desirable because they would have favored poor women ^34^ ^,^ ^35^.

The procedure for estimating the concentration index and the methodology for its decomposition are described elsewhere ^34^. Briefly, a logistic regression model was first estimated, with continuum of care-maternal health as the outcome variable and women’s and household sociodemographic characteristics as explanatory variables. The concentration index was then decomposed as the weighted sum of the marginal effects of each variable estimated in the model, where the weights corresponded to the concentration indices of the explanatory variables themselves (Supplementary Material; https://cadernos.ensp.fiocruz.br/ojs/index.php/csp/article/view/11713/21036). All calculations took into account the complex survey design and were performed in Stata, version 15.0 (https://www.stata.com).

## Results

### Description of the population

The sample analyzed consisted of 2,846 women who had a live birth within the five years preceding each survey wave. Of these, 15.2% were adolescents (95% confidence interval - 95%CI: 13.6-16.9), and 84.8% (95%CI: 83.1-86.4) adults. Most had at least secondary schooling (87.8%, 95%CI: 86.0-89.4), were living with a partner (78.7%, 95%CI: 76.5-80.7), and lacked Social Security coverage (63.5%, 95%CI: 60.5-66.4). Most were non-Indigenous (92.2%, 95%CI: 90.1-93.8) and resided in urban areas (76.2%, 95%CI: 74.0-78.4). One-third were primiparous (32.4%, 95%CI: 30.1-34.7), and 40.6% (95%CI: 37.7-43.6) were from the poorest tertile (Table 1).

**Table 1:** Sociodemographic and household characteristics of women with a live-born child in the five years preceding the survey. *Mexican National Continuous Health and Nutrition Survey* (ENSANUT), Mexico, 2021-2023.

Characteristics	Total (N = 2,846; 100%)		
	n	%	95%CI
Sociodemographic characteristics			
Maternal age at birth			
Adolescent	463	15.2	13.6-16.9
Adult	2,383	84.8	83.1-86.4
Levels of education			
Primary or less	410	12.2	10.6-14.0
Secondary or higher	2,436	87.8	86.0-89.4
Marital status			
Without partner	740	21.3	19.3-23.5
With partner	2,106	78.7	76.5-80.7
Primiparity			
Yes	902	32.4	30.1-34.7
No	1,944	67.6	65.3-69.9
Health insurance			
No Social Security *	1,894	63.5	60.5-66.4
Social Security **	952	36.5	33.6-39.5
Household-level characteristics			
Socioeconomic status			
Tertile 1	1,155	40.6	37.7-43.6
Tertile 2	970	29.8	27.4-32.3
Tertile 3	721	29.6	26.9-32.4
Indigenous			
Yes	210	7.8	6.2-9.9
No	2,636	92.2	90.1-93.8
Place of residence			
Rural	830	23.8	21.6-26.0
Urban	2,016	76.2	74.0-78.4
Year of birth of the last live-born child			
Pandemic ***	1,300	47.9	45.0-50.8
Pre-pandemic ^#^	1,546	52.1	49.2-55.0

95%CI: 95% confidence interval.

Note: the estimates considered the complex survey design.

* Includes women who are not affiliated with or enrolled in medical services, as well as those affiliated with IMSS-Well-being (IMSS-B) (formerly Oportunidades), Institute of Health for Well-being (INSABI), and those who reported having Seguro Popular in 2018;

** Social Security includes: Mexican Social Security Institute (IMSS), Institute for Social Security and Services for State Workers (ISSSTE), Petróleos Mexicanos (PEMEX), and the Social Security Institute for the Armed Forces;

*** It considers births that occurred in the five years preceding the survey and takes into account the official date of the pandemic declaration in Mexico (24 March 2020 to 9 October 2023);

# It considers births that occurred in the five years prior to the pandemic declaration (11 August 2016 to 23 March 2020).

### Independent vs. conditional coverage

Table 2 shows that among the independent coverage indicators, the lowest percentages were found in timely antenatal care, 61.8% (95%CI: 59.0-64.6), antenatal care with adequate content, 79.5% (95%CI: 77.0-81.7), and institutional childbirth, 81.1% (95%CI: 78.2-83.7). The highest values were observed for qualified antenatal care, 98.2% (95%CI: 97.3-98.8), and postpartum care, 90.8% (95%CI: 88.7-92.5). However, when two or more interventions were considered, conditional coverage decreased as the number of required interventions increased, such that continuum of care-maternal health was provided to only 37.3% of women (95%CI: 34.6-40.1).

**Table 2:** Independent and conditional coverage indicators of the continuum of care for maternal health by socioeconomic status. *Mexican National Continuous Health and Nutrition Survey* (ENSANUT), Mexico, 2021-2023.

Coverage indicators	Total (N = 2,846) *		Socioeconomic status						p-value
			Tertile 1 (n = 1,155)		Tertile 2 (n = 970)		Tertile 3 (n = 721)		
	%	95%CI	%	95%CI	%	95%CI	%	95%CI	
Independent									
Skilled antenatal care	98.2	97.3-98.8	96.7	94.6-97.9	99.2	98.3-99.6	99.4	98.1-99.8	< 0.001
Timely antenatal care	61.8	59.0-64.6	51.9	48.1-55.7	65.9	60.9-70.5	71.4	66.1-76.2	< 0.001
Frequent antenatal care	86.2	84.2-88.0	80.3	76.6-83.5	87.9	84.6-90.6	92.6	87.8-95.6	< 0.001
Adequate antenatal care	79.5	77.0-81.7	72.1	68.0-76.0	82.2	78.3-85.6	86.7	81.6-90.6	< 0.001
Institutional childbirth	81.1	78.2-83.7	86.0	82.3-89.1	82.7	78.6-86.2	72.8	67.0-77.9	< 0.001
Postpartum care	90.8	88.7-92.5	90.8	87.7-93.1	90.7	87.7-93.1	90.7	85.4-94.3	0.9995
Conditional									
Skilled antenatal care	98.2	97.3-98.8	96.7	94.6-97.9	99.2	98.3-99.6	99.4	98.1-99.8	< 0.001
Skilled and timely antenatal care	61.5	58.7-64.3	51.5	47.7-55.3	65.8	60.9-70.5	71.0	65.6-75.8	< 0.001
Skilled, timely and frequent antenatal care	57.7	54.8-60.4	46.9	42.8-51.1	63.2	58.4-67.8	66.7	60.9-72.1	< 0.001
Skilled, timely, frequent and adequate antenatal care	48.7	46.1-51.4	37.5	33.5-41.7	53.3	48.6-58.0	59.5	53.8-65.0	< 0.001
Skilled, timely, frequent and adequate antenatal care, and institutional childbirth	39.5	36.7-42.3	34.0	30.2-38.0	44.2	39.6-48.9	42.2	36.6-48.0	< 0.005
Continuum of care **	37.3	34.6-40.1	32.9	29.1-36.9	40.9	36.3-45.6	39.7	34.2-45.5	< 0.05

95%CI: 95% confidence interval.

Note: the estimates considered the complex survey design.

* Women covered by private health insurance schemes were excluded due to their low representation (n = 5);

** Includes skilled, timely, frequent, and adequate antenatal care, institutional delivery, and at least one consultation within the first week after birth.

An analysis of the association between independent coverage indicators and socioeconomic status revealed disparities at each antenatal care phase, with women from tertiles 2 and 3 consistently showing higher prevalence rates than those from the poorest tertile. This pattern was not observed in institutional childbirth, where coverage was higher among women from the poorest tertile (86%, 95%CI: 82.3-89.1) than among those in tertile 3 (72.8%, 95%CI: 67.0-77.9).

Significant differences (p < 0.05) were observed by socioeconomic status across all indicators in the conditional coverage of antenatal care. Among pregnant women in the poorest tertile, 37.5% (95%CI: 33.5-41.7) received qualified, timely, frequentand adequate antenatal care, compared with 59.5% (95%CI: 53.8-65.0) of those in the highest tertile. Childbirth care followed a similar pattern, although with smaller differences between groups: coverage of qualified, timely, and frequent antenatal care with adequate content, combined with institutional childbirth, came to 34% (95%CI: 30.2-38.0) among the poorest women, vs. 42.2% (95%CI: 36.6-48.0) for those in tertile 3. As a result, only 32.9% (95%CI: 29.1-36.9) of women in the poorest tertile received continuum of care-maternal health compared with 39.7% (95%CI: 34.2-45.5) for those in the highest tertile.

### Prevalences and factors associated with receiving continuous care

At the national level, the prevalence of continuum of care-maternal health was 37.3% (95%CI: 34.55-40.1). Coverage was notably higher among women with at least secondary education, those affiliated with Social Security, and those belonging to tertiles 2 and 3, with prevalence estimates of 38.5% (95%CI: 35.5-41.6), 43.5% (95%CI: 38.5-48.6), 40.9% (95%CI: 36.4-45.4) and 39.7% (95%CI: 34.2-45.4), respectively. Similarly, non-Indigenous women (38.7%, 95%CI: 35.8-41.6) and those who gave birth during the COVID-19 pre-pandemic period (43.9%, 95%CI: 40.1-47.7) showed higher levels of coverage. By contrast, substantially lower prevalence was observed among women with less than secondary education, without Social Security affiliation, and those in the poorest tertile (28.5%, 95%CI: 22.5-35.4; 33.7%, 95%CI: 30.7-36.8; and 32.8%, 95%CI: 29.0-36.9, respectively). The lowest coverage occurred among Indigenous women (21%, 95%CI: 13.6-31.0) and among those who gave birth during the pandemic (30.1%, 95%CI: 26.2-34.4) (Table 3).

**Table 3:** Prevalences and sociodemographic characteristics of women associated with the continuum of care for maternal health. *Mexican National Continuous Health and Nutrition Survey* (ENSANUT), Mexico, 2021-2023 .

Characteristics	Received continuum of care * [n = 1,117 (37.3%)]		p-value	Adujsted OR	95%CI	p-value
	%	95%CI				
Sociodemographic characteristics						
Maternal age at birth			0.243			0.610
Adolescent	34.2	29.3-39.4		1.00		
Adult	37.8	34.7-41.1		1.30	0.8-1.5	
Levels of education			< 0.05			0.110
Primary or less	28.5	22.5-35.4		1.00		
Secondary or higher	38.5	35.5-41.6		1.33	0.9-1.9	
Marital status			0.171			0.214
Without partner	34.1	29.1-39.5		1.00		
With partner	38.2	35.1-41.3		1.19	0.7-1.3	
Primiparity			0.824			0.979
Yes	36.9	32.8-41.1		1.00		
No	37.5	34.1-41.0		1.00	0.7-1.3	
Health insurance			< 0.01			< 0.05
No Social Security **	33.7	30.7-36.8		1.00		
Social Security ***	43.5	38.5-48.6		1.37	1.1-1.8	
Household-level characteristics						
Socioeconomic status			< 0.05			
Tertile 1	32.8	29.0-36.9		1.00		
Tertile 2	40.9	36.4-45.4		1.19	0.9-1.6	0.236
Tertile 3	39.7	34.2-45.4		1.02	0.7-1.4	0.897
Indigenous			< 0.01			< 0.01
Yes	21.0	13.6-31.0		1.00		
No	38.7	35.8-41.6		2.08	1.2-3.6	
Place of residence			0.260			0.550
Rural	34.7	30.0-39.7		1.00		
Urban	38.1	34.9-41.4		0.92	0.7-1.2	
Year of birth of the last live-born child			< 0.001			< 0.001
Pandemic ^#^	30.1	26.2-34.4		1.00		
Pre-pandemic ^##^	43.9	40.1-47.7		1.84	1.4-2.4	

95%CI: 95% confidence interval.

Note: the estimates considered the complex survey design.

* Includes skilled, timely, frequent, and adequate antenatal care, institutional delivery, and at least one consultation within the first week after birth;

** Includes women who are not affiliated with or enrolled in medical services, as well as those affiliated with IMSS-Well-being (IMSS-B) (formerly *Oportunidades*), Institute of Health for Well-being (INSABI), and those who reported having *Seguro Popular* in 2018;

*** Social Security includes: Mexican Social Security Institute (IMSS), Institute for Social Security and Services for State Workers (ISSSTE), Petróleos Mexicanos (PEMEX), and the Social Security Institute for the Armed Forces;

^#^ It considers births that occurred in the five years preceding the survey and takes into account the official date of the pandemic declaration in Mexico (24 March 2020 to 9 October 2023);

^##^ It considers births that occurred in the five years prior to the pandemic declaration (11 August 2016 to 23 March 2020).

Our statistical model showed that continuum of care-maternal health was more likely among women affiliated with Social Security (odds ratio - OR = 1.37, 95%CI: 1.1-1.8), those whose children were born during the pre-pandemic period (OR = 1.84, 95%CI: 1.4-2.4), and non-Indigenous women (OR = 2.08; 95%CI: 1.2-3.6), relative to their respective reference groups. Moreover, women with at least secondary education were 33% (95%CI: 0.9-1.9), more likely to receive continuum of care than those with primary schooling or less although this association was p = 0.11 (Table 3).

### Estimation and decomposition of inequality

The Wagstaff concentration index for continuum of care-maternal health was 0.098 (p < 0.05), indicating a disproportionate concentration among women of higher socioeconomic status (Table 4). All variables had positive concentration index (column 6), except non-primiparity, which showed that a greater proportion was found among women in more favorable socioeconomic circumstances. The main contributors to this inequality were Social Security affiliation (31.1%), non-Indigenous status (30.03%), secondary or higher education (16.07%), and pre-pandemic period (9.6%) (column 9). The model explained 92.02% of the variation in the concentration index.

**Table 4:** Decomposition of inequality in the continuum of care for maternal health by women’s sociodemographic characteristics. *Mexican National Continuous Health and Nutrition Survey* (ENSANUT), Mexico, 2021-2023.

Explanatory variables	Marginal efect	p-value	Proportion	Elasticity	Concentration index	p-value	Concentration index	Relative contribution (%)
Sociodemographic characteristics								
Maternal age at birth								
Adolescent								
Adult	0.018	0.62	0.848	0.041	0.035	< 0.01	0.003	2.41
Levels of education								
Primary or less								
Secondary or higher	0.064	0.11	0.878	0.152	0.065	< 0.01	0.015	16.07
Marital status								
Without partner								
With partner	0.039	0.21	0.787	0.083	0.005	0.588	0.001	0.64
Primiparity								
Yes								
No	0.000	0.98	0.676	0.001	-0.010	0.349	0.000	-0.02
Health insurance								
No Social Security *								
Social Security **	0.070	< 0.05	0.365	0.069	0.275	< 0.01	0.030	31.10
Household-level characteristics								
Socioeconomic status								
Tertile 1								
Tertile 2	0.039	0.23	0.298	0.031	0.110	< 0.01	0.005	5.62
Tertile 3	0.004	0.90	0.295	0.004	0.703	< 0.01	0.004	4.45
Indigenous status								
Yes								
No	0.163	< 0.01	0.922	0.405	0.045	< 0.01	0.029	30.03
Place of residence								
Rural								
Urban	-0.018	0.55	0.762	-0.038	0.125	< 0.01	-0.007	-7.82
Year of birth of the last live-born child								
Pandemic ***								
Pre-pandemic ^#^	0.136	< 0.001	0.521	0.191	0.031	0.058	0.009	9.60
Explained inequality							0.090	92.02
Residual							0.008	
Estimated inequality							0.098	

Note: the estimates considered the complex survey design.

* Includes women who are not affiliated with or enrolled in medical services, as well as those affiliated with IMSS-Well-being (IMSS-B) (formerly *Oportunidades*), Institute of Health for Well-being (INSABI), and those who reported having *Seguro Popular* in 2018;

** Social Security includes: Mexican Social Security Institute (IMSS), Institute for Social Security and Services for State Workers (ISSSTE), Petróleos Mexicanos (PEMEX), and the Social Security Institute for the Armed Forces;

*** It considers births that occurred in the five years preceding the survey and takes into account the official date of the pandemic declaration in Mexico (24 March 2020 to 9 October 2023);

^#^ It considers births that occurred in the five years prior to the pandemic declaration (11 August 2016 to 23 March 2020).

## Discussion

The findings of this study demonstrate significant socioeconomic inequalities in the continuum of care (continuum of care) for maternal health (maternal health), favoring women from better-off socioeconomic backgrounds. Women in the poorest tertile were markedly less likely to obtain comprehensive continuum of care than those with greater economic means. Moreover, our analysis enabled the identification of the key factors underlying these inequalities, including ethnicity, health insurance scheme, maternal education level, and the birth period of the child (vis-à-vis the COVID-19 pandemic). Finally, the study quantified the relative contribution of each factor to the observed inequality.

The levels achieved in continuum of care-maternal health coverage can be better understood by analyzing each of the independent coverage indicators comprised in the maternity cycle, particularly timely antenatal care, which showed a significant lag in recent years compared to levels previously reported ^4^ ^,^ ^15^. One notable finding concerns institutional childbirth. In contrast to the general pattern of increased coverage among women from the higher tertile, the greatest coverage occurred among women from the poorest tertile. This unexpected result may have been influenced by the way response categories for the birth-care site were defined across the survey waves. In some cases, it was impossible to distinguish whether births took place in private hospitals - facilities more commonly utilized by women with greater economic means.

Conditional coverage indicators revealed substantial gaps by socioeconomic status in timely, frequent, and adequate antenatal care. This aligns with previous research highlighting a gap in this care that has worsened among vulnerable groups. Consequently, only a small proportion of women benefit from the continuum of care-maternal health, and these are predominantly women from more favorable socioeconomic backgrounds ^13^ ^,^ ^15^.

Our findings also document that being Indigenous is a key factor associated with a lower likelihood of receiving continuum of care-maternal health and contributes significantly to inequality decomposition. This result was expected, given that Indigenous populations have long experienced more precarious living conditions than the general population and have historically faced profound structural and social disadvantages. These inequalities have limited their access to Social Security health services, basic services, quality food, and housing ^36^, problems that worsened during COVID-19 ^37^ ^,^ ^38^. Unfortunately, a discriminatory ideology persists in Mexico, reflected in normalized practices of exclusion and mistreatment across various spheres of social life ^39^. This situation has generated distrust of the dominant health system among some Indigenous communities, thereby severely limiting accessibility and the quality of maternal health care ^40^. Reducing these inequalities among highly vulnerable populations such as Indigenous communities requires coordinated strategies on both the supply and demand sides ^41^. On the supply side (health services), there is a need to strengthen infrastructure and the availability of essential inputs, implement mandatory training for personnel in human rights and cultural competence, integrate traditional midwives, and ensure linguistically accessible services ^42^. On the demand side, interventions aimed at reducing economic barriers for the population - such as targeted subsidies for obstetric transport, accommodation, and culturally appropriate support - have been shown to be effective ^41^ ^,^ ^43^ ^,^ ^44^.

At the national level, the total proportion of women receiving the continuum of care-maternal health (37.3%) was 16-percentage-point lower than that reported in a study conducted in 2015-2018 ^4^. This reduction may stem from recent reforms in the health subsystem serving the population without access to Social Security. The changes may have made it more difficult for this population to recognize and exercise their right to health services, thereby contributing to reduced access within public care. According to recent studies, the number of households lacking access to such services increased from 16% of the total population (20 million people) in 2018 to 28% (35.7 million) in 2020 ^45^, with the most affected households being those that were poorest, located in Indigenous communities, headed by women, or with low education levels ^13^ ^,^ ^45^. Access to maternal health services was further constrained by measures implemented during the COVID-19 pandemic, including home isolation due to the suspension of economic activities and services, as well as hospital conversions that limited the provision of non-COVID-19 care ^24^ ^,^ ^46^. Collectively, these factors explain the decline in maternal health coverage.

In health systems characterized by segmentation and fragmentation, such as Mexico’s, the existence of different insurance schemes for distinct population groups according to their position in the labor market contributes to the persistence of inequalities ^20^ ^,^ ^21^. This is driven by disparities in public health expenditure per beneficiary and the package of services provided, together with differences in the professional profiles and income levels of those with and without Social Security coverage ^20^ ^,^ ^21^ ^,^ ^47^. The population without Social Security coverage, served by the federal and state Ministries of Health, receives a smaller share of health expenditure and systematically faces greater barriers to care than the population affiliated with Social Security. Pregnant women without Social Security coverage generally have lower income and education levels, are self-employed or work in the informal sector, and reside in marginalized areas ^3^ ^,^ ^11^ ^,^ ^14^ ^,^ ^21^, thereby facing fewer opportunities to attain the continuum of care-maternal health ^4^.

This finding underscores the importance of implementing proactive outreach strategies, such as mobile brigades and home-based follow-up, particularly in rural areas, to improve access for maternal health care ^48^. Additionally, reducing these inequalities will require structural reforms in the Mexican health system that progressively advance functional unification, supported by increased public financing and stronger service-sharing across institutions, aimed at equalizing per capita funding for all beneficiaries ^49^.

On the other hand, the pattern observed for women’s education level is noteworthy. Although this variable accounted for approximately 16% of the decomposition of inequality in continuum of care-maternal health, it did not reach statistical significance in either the logistic regression model or the inequality analysis. This finding contrasts with previous evidence showing greater continuity in continuum of care-maternal health among women with higher levels of education, as well as the contribution of education to inequalities in continuity of care ^5^ ^,^ ^9^ ^,^ ^10^ ^,^ ^50^. One possible explanation lies in the relative weight of indigenous status and Social Security affiliation in the estimation of inequalities. Within the context analyzed, women with lower levels of education are disproportionally concentrated among the indigenous population and among those without access to Social Security ^51^ ^,^ ^52^, which may have attenuated the independent effect of education in the models. Future research should examine these mechanisms in greater detail and explore how education, Indigenous status, and access to Social Security interact to shape inequalities in maternal health care.

This study had some limitations. First, due to its cross-sectional design, recall bias may have affected the accuracy of the data and limited the ability to infer causal relationships between variables. Second, since the data collection tools were not specifically designed to study maternal health care, the estimation of inequality in continuum of care-maternal health may be underestimated. This may be due to the lack of information on variables that can constitute barriers to the use of health services, such as travel and waiting times, as well as service hours. Moreover, the context of the COVID-19 pandemic may have reduced maternal health care coverage through changes in pregnant women’s care-seeking patterns driven by isolation measures or fear of infection, and reduced service supply.

Other potentially influential factors associated with the use of the continuum of care-maternal health - e.g., maternal health information, women’s autonomy and decision-making power, freedom of movement, and previous pregnancy experience - were also beyond the scope of this study. Finally, it was challenging to accurately record the place of birth for women treated in private hospitals, due to changes in response categories across survey waves. This may have led to underestimation of this indicator, with birth-care coverage primarily reflecting public hospital data. In turn, the overall continuum of care-maternal health may also have been underestimated. Despite these limitations, the present study provides evidence to help identify interventions that need strengthening across the continuum of care-maternal health, as well as key factors that should be considered in the design and implementation of policies to reduce inequalities in access to comprehensive maternal health care. Although the analysis focuses on Mexico, the findings are relevant to other countries facing similar structural inequalities in the provision of maternal health services.

## Conclusions

Despite recent public health policy efforts, progress in coverage of the continuum of care for maternal health in Mexico remains insufficient, hindering advancement toward universal health coverage, as outlined in target 3 of the 2030 Sustainable Development Goals. Marked inequalities in the utilization of maternal health services persist, associated with structural disparities that place women of lower socioeconomic and education levels, Indigenous women, and those without Social Security coverage at a social disadvantage. Reducing these gaps requires intersectoral policies that address key social determinants of health, such as education, employment, and access to quality public services, particularly among socially and economically vulnerable groups. At the same time, it is necessary to strengthen the health system by a greater and more equitable allocation of resources, regardless of the subsystem to which individuals are affiliated, ensuring access to quality services and the effective exercise of the right to health for all women. Within this process, strategies that prioritize women’s empowerment and equality are essential, particularly for addressing the role of gender in shaping access to and utilization of health services.

## Data Availability

The sources of information used in the study are indicated in the body of the article.

## Material Suplementar

Material Suplementar
