# Supplementary material for: Socioeconomic inequalities in the continuum of care for maternal health in an upper-middle-income country: the case of Mexico
Source: Cad Saude Publica. 2026 Aug 21;42:e00232925. doi: 10.1590/0102-311XEN232925 (PMC13505640; doi:10.1590/0102-311XEN232925)
Supplement: Supplementary file 1 [file 1678-4464-csp-42-EN232925-s.pdf]

Supplementary material

Socioeconomic inequalities in the continuum of care for maternal health in an upper-middle-income country: The case of Mexico

Decomposition of inequality of concentration index the continuum of care for maternal health

In this study, we used the concentration index (CI)[1,2] to estimate inequality along the continuum of care for maternal health (CoC-MH) according to socioeconomic statue (SES).

For any outcome variable *h* (in this case, CoC-MH), the concentration index, CI(h), was calculated as follows (1,2):

$$CI(h)=\frac{2}{\mu}*\mathrm{cov}(h_i,r_i)\,(1)$$

where h_i was the value of the variable for individual i, r_i was the fractional rank of individual i once the population was ranked according to their SES, and μ was the expected value (mean) of h. Since CoC-MH was an indicator variable, we used the CI proposed by Wagstaff for binary variables:

$$WCI(h)=\frac{CI(h)}{1-\mu}\,(2)$$

where CI was obtained from (1).

Decomposition of inequality

The concentration index CI(*h*) was decomposed into k explanatory factors as follows:

$$CI(h)=\sum_k\left(\frac{\beta_k^m\,\bar{x}_k}{\mu}\right)CI_k+GCI_\varepsilon\,(3)$$

where β_k^m represented the partial effects estimated through a generalised linear model for variable h and evaluated at the sample means, $\bar{x}_k$ was the mean of each explanatory variable k, CI_k was the CI for each variable x_k, and GC_ε was the generalised CI for the error term (ε) and reflected the SES-related inequality not explained by the model, which should approach zero in a well-specified model.

Applying (2) in (3), we had the following decomposition for the Wagstaff CI:

$$WCI(h)=\frac{\sum_k \left(\frac{\beta_k^m \bar{x}_k}{\mu}\right) CI_k}{1-\mu}+\frac{GCI_\varepsilon}{1-\mu} \quad (4)$$

We employed the CI (2) to estimate inequality in the CoC-MH and used the previously described logistic model (4) to estimate partial effects for inequality decomposition.

References

1. Wagstaff A. The bounds of the concentration index when the variable of interest is binary, with an application to immunization inequality. Health Econ. 2005;14(4):429-32. doi: 10.1002/hec.953.
2. O'Donnell O, van Doorslaer E, Wagstaff A, Lindelow M. Analyzing Health Equity Using Household Survey Data. Analyzing Health Equity Using Household Survey Data. The World Bank; 2007.
